# Herbal medicine for Behcet's disease: A protocol for a systematic review and meta-analysis

**DOI:** 10.1097/MD.0000000000010165

**Published:** 2018-03-30

**Authors:** Ji Hee Jun, Tae-Young Choi, Junhua Zhang, Mi Mi Ko, Myeong Soo Lee

**Affiliations:** a Clinical Research Division, Korea Institute of Oriental Medicine; b Department of Preventive Medicine, College of Korean Medicine, Daejeon University, Daejeon, Republic of Korea; c Evidence Based Medicine Center, Tianjin University of Traditional Chinese Medicine, Tianjin, China; d Department of Korean Medicine Life Science, Korea University of Science & Technology, Daejeon, Republic of Korea.

**Keywords:** Behcet's disease, herbal medicine, systematic review

## Abstract

**Background:**

Herbal medicine treatment is often recommended in order to raise immunity levels and reduce the possibility of recurrence of symptoms, and treating the fundamental causes of the disease. This systematic review will assess the efficacy of herbal medicine in treating Behcet's disease.

**Methods and analysis:**

We will search the following 11 electronic databases from their inception: PubMed, EMBASE, and CENTRAL; 3 Chinese databases (CNKI, Wanfang, and VIP); and 5 Korean databases (OASIS, DBpia, RISS, KISS, and KoreaMed). The data will be extracted independently by 2 authors using predefined criteria. Disagreements will be resolved by discussion between the authors. The risk of bias will be assessed using the Cochrane tool for assessing risk of bias.

**Ethics and dissemination:**

Ethical approval will not be required, given that this protocol is for a systematic review. The review will be published in a journal. Updates of the review will be conducted periodically to inform and guide healthcare practice and policy.

**Trial registration number:**

PROSPERO 2018 CRD42018085493

## 1 Introduction

Behcet's disease (BD), also known as Behcet's syndrome, is a chronic inflammatory disease that can affect the skin, blood vessels, gastrointestinal tract, central nervous system, heart, and other organs, as well as causing canker sores, genital ulcers, and eye inflammation. The basic feature of each symptom is body-wide angitis, (blood vessel inflammation).^[1,2]^ It tends mostly to affect people in their 20s and 30s, and symptoms can gradually disappear over time.^[3]^

While the cause of Behcet's disease remains unknown, it may be an autoimmune disorder, involving both genetic predisposition and environmental factors. In particular, a gene called *HLA-B51*, which is known to be present in patients suffering from Behcet's disease, may be significant.^[4,5]^ Conventional medicine commonly treats Behcet's disease by means of immunosuppressive, anti-inflammatory drugs and steroid agents. While the effects are rapid, with proven efficacy, this treatment can negatively impact patient immunity if administered over long periods of time, risking recurrence of symptoms and the possibility of complications and adverse effects.^[6,7]^

Accordingly, herbal medicine treatment is often recommended in order to raise immunity levels and reduce the possibility of recurrence of symptoms, as well treating the fundamental causes of the disease.^[8]^ Herbal medicine slowly heals the body over a long period of time, relieving patients of ulcers and helping to restore the immune system.^[8,9]^ Ultimately, this reduces the likelihood of recurrence and helps patients to recover full health, with fewer adverse effects than treatment informed by conventional medicine.^[10,11]^

Although there are existing systematic reviews addressing the use of herbal medicine for treatment of Behcet's disease,^[12–16]^ the proposed study will undertake further updated research to establish the safety and efficacy of herbal medicine in the treatment of Behcet's disease.

## 2 Methods

### 2.1 Study registration

This protocol review has been registered on PROSPERO 2018 CRD42018085493 (Available from: http://www.crd.york.ac.uk/PROSPERO/display_record.php?ID=CRD42018085493).

### 2.2 Data source

Eleven databases will be searched from their inception to March 2018: PubMed, EMBASE and CENTRAL; 3 Chinese databases (CNKI, Wanfang, and VIP); and 5 Korean databases (OASIS, DBpia, RISS, KISS, and Korea Med). We will use the following search terms: (BD OR Behcet's syndrome OR Adamantiades-Behcet's Syndrome OR Hulusi-Behcet's syndrome OR Ouclo-bucco-genital syndrome OR Touraine's aphthosis OR Triple Symptom Complex of Behcet) AND (traditional Chinese medicine OR Korean medicine OR kampo medicine OR traditional medicine OR herbal medicine OR decoction). Searches will be conducted in Korean, English, and Chinese.

### 2.3 Study selection

#### 2.3.1 Types of studies

We will include all randomized controlled trials (RCTs) or quasi-RCTs comparing decoction types of herbal medicine with conventional medicine. Case studies, qualitative studies, uncontrolled trials, and reviews will be excluded, as will trials that fail to provide detailed results.

#### 2.3.2 Types of participants

Participants of both sexes and of any age with clinically diagnosed Behcet's disease will be included, provided they have been diagnosed according to International Study Group (ISG) criteria,^[17]^ which are primary symptoms of Behcet's disease plus any 2 of the following 4 symptoms: recurrent genital ulcerations; eye inflammation; skin lesions; positive pathergy test or International criteria for Behcet's Disease.^[18]^

#### 2.3.3 Types of intervention

Only decoction types of herbal medicine will be included, with no limitations on number, administration method, dosage, or duration of treatment. Comparators will include conventional medicine or no treatment or placebo. We will exclude trials using other types of herbal medicine for comparison, or those using a different baseline therapy.

#### 2.3.4 Types of outcome measurements

*Primary outcomes*:

1. Response rate of symptom reduction
2. Recovery rate

*Secondary outcomes*:

1. Laboratory changes in C-reactive protein (CRP), erythrocyte sedimentation rate (ESR)
2. Mucocutaneous manifestations (oral ulcer, genital ulcer, erythema nodosum): frequency, number, duration, severity
3. Adverse events (AEs).

### 2.4 Data extraction and assessment of risk of bias

#### 2.4.1 Data extraction

All studies from the electronic database searches will be reviewed by JHJ and MMK. Two authors (TYC and JZ) will select the relevant trials by reviewing titles and abstracts. Any disagreements will be resolved by discussion between the 2 authors and an arbiter (MSL), and authors of the included trials will be contacted for clarification if necessary. Data will be collected from the included trials by 2 authors (JHJ and MMK). The extracted data will include specific details about the author name(s), year of publication, sample size, age and sex of participants, intervention (regimen), control (regimen), main outcomes, and adverse effects.

#### 2.4.2 Assessment of risk of bias

We will independently assess the risk of bias as described in the Cochrane Handbook for Systematic Reviews of Interventions.^[19]^ The following items will be assessed: random sequence generation; allocation concealment; blinding; incomplete outcome data; selective outcome reporting; other bias. In case of any unresolved disagreements, MSL will make the final decision as arbiter.

### 2.5 Data analysis

All statistical analyses will be performed using Review Manager software (version 5.3). Dichotomous data will be reported as risk ratio with 95% confidence intervals (CIs), and continuous data will be reported as mean difference with 95% CIs. For primary outcomes, if the meta-analysis results are significantly heterogeneous, subgroup analysis will be performed as detailed below.

We will use the Grading of Recommendations Assessment, Development and Evaluation (GRADE) software to determine the quality of evidence based on the Cochrane Handbook for Systematic Reviews of Interventions to create a Summary of Findings table.

#### 2.5.1 Assessment of heterogeneity

We will use the random effect model for meta-analysis. If a meta-analysis is possible, we will use the *I*^2^ statistic to quantify inconsistencies across the included studies. A resulting 50% cut-off point would represent substantial heterogeneity. Where heterogeneity is exited, we will conduct subgroup analyses.

#### 2.5.2 Subgroup analysis and investigation of heterogeneity

Where studies and data permit, subgroup analyses will be conducted according to the kind of herbal medicine or duration of treatment.

#### 2.5.3 Sensitivity analysis

We will use sensitivity analyses to investigate suspected funnel plot asymmetry according to the following criteria:

1. Methodological qualities (sequence generation, allocation concealment, or blinding in the assessment of outcomes and symptom severity);
2. Sample size (more or less than 30 participants in each group).

#### 2.5.4 Assessment of reporting biases

If more than 10 studies are available, we will assess funnel plot asymmetry for publication bias and small study effects using Egger's method.^[20]^ As funnel plot asymmetry does not necessarily indicate publication bias, we will attempt to distinguish possible reasons for the asymmetry, including poor methodological quality and true heterogeneity of studies.

### 2.6 Ethics and dissemination

Ethical approval is not required, given that this protocol is for a systematic review. The findings of this review will be disseminated widely through peer-reviewed publications and conference presentations.

## 3 Discussion

As this is a systematic review, there is no primary data collection in relation to BD. The updated review will provide a summary of the current state of evidence regarding the effectiveness of decoction types of herbal medicine in managing BD. The review will be updated periodically to inform and guide healthcare practice and policy.

## Author contributions

**Conceptualization:** J.H. Jun.

**Data curation:** J.H. Jun, T-Y. Choi.

**Formal analysis:** J.H. Jun, M.M. Ko.

**Funding acquisition:** M.S. Lee.

**Investigation:** M.S. Lee.

**Methodology:** J. Zhang, M.M. Ko, M.S. Lee.

**Supervision:** M.S. Lee.

**Validation:** M.S. Lee, T-Y. Choi.

**Writing – original draft:** J.H. Jun, M.M. Ko, M.S. Lee.

**Writing – review & editing:** J. Zhang, T-Y. Choi.
